# Items

**Published:** 1882-01

**Authors:** 


### Items.

Dr. Thomas W. Grimes died suddenly, of disease of the heart, at his home in Columbus, on December 12, in the seventy-third year of his age.

Dr. Grimes was born in Greensboro, Ga., August 18,

1809. He received his degree of Doctor of Medicine from the Medical College of Geórgia, at Augusta, being a member of the first class that ever graduated at that institution. He practiced his profession in the place of his nativity until 1849, when he removed to Columbus, where he has since resided, actively engaged, to the hour of his death, in the discharge of the duties which devolved upon him as a physician. He has been a member of the Medical Association of Georgia since 1855.

Dr. Grimes had many friends throughout the State, to whom the announcement of his death brought a pang of sincere regret. He will be greatly missed in the community where, for more than thirty years, he has led a quiet, unostentatious and useful life, exemplifying in his daily walk the true Christian, the faithful friend, the loyal citizen and the kind physician.

He was interred in Columbus, by the side of his wife, who preceded him to the grave only a few months.

---

For several weeks the water in certain parts of Boston has had a disgusting taste and very offensive odor, not unlike that of fish oil.

Upon investigation the cause has been discovered to be the fresh-water sponge, which was found growing abundantly in one of the ponds from which the water is obtained. This sponge is generally arborescent in shape, commonly under a foot in length, of a green color when alive, and prospers in the bottom of filthy ponds—though clean ponds are frequently invaded by it.

---

The burning of the Ohio State Institution for Idiotic and Imbecile Youths, located near Columbus, should serve as a timely warning to those in charge of similar institutions throughout the country. Fortunately, the fire occurred in the daytime, otherwise a heart-rending calamity—the death of many of these unfortunates—would have been the inevitable result. The building, valued at $200,000, was totally destroyed. As usual, the water supply was insufficient, and the fire apparatus was found utterly worthless.

---

In view of the prevalence of small-pox in certain northern and western cities, the city council of Atlanta, upon the recommendation of the board of health, has appropriated $1,000 to be expended, under the direction of the board, to secure a thorough vaccination of the people. The board has adopted a plan of house-to-house visitation, urging upon the people to accept the benefits freely offered, and has also established a central dispensary for the gratuitous vaccination of any persons who may apply. The undertaking has been committed to Dr. William Henry Cumming, of this city, who, with an ample corps of assistants, will vigorously prosecute the work.

---

The registry of medical practitioners in this, Fulton county, contained on the first day of December, 111 names. Sixteen have since been added. Of these, 115 reside and practice in Atlanta—115 doctors to 40,000 people; and some, it is likely, have failed to register. There certainly ought to be no lack of medical advice in this community.

We refer to this subject, not for the purpose of retarding the immigration of M. D.'s, but merely as an item of news.

---

Sir James Paget is ill with pneumonia. The attack is similar to other seizures he has suffered from since he was laid up with blood poisoning. As soon as he is able to travel, he will be removed to the south of Europe, where he will probably spend the winter.

The condition of this distinguished gentleman's health is a cause for much anxiety to his friends.

---

On the first day of December, not a single practitioner of medicine had registered in Chatham county, and the clerk of the court had not provided the book required by law.

That clerk would surely be entitled to the stakes in a slow race. For slowness he can scarcely be beaten.

